# P-1339. In vitro activity of ceftazidime-avibactam and comparators against Pseudomonas aeruginosa collected worldwide stratified by age group, ATLAS Global Surveillance Program, 2019-2023

**DOI:** 10.1093/ofid/ofaf695.1527

**Published:** 2026-01-11

**Authors:** Meredith Hackel, Gregory Stone, Katherine Perez, Paurus Irani, Daniel F Sahm

**Affiliations:** IHMA, Schaumburg, IL; Pfizer, Inc., Groton, Connecticut; Pfizer, Inc., Groton, Connecticut; Pfizer United Kingdom, London, England, United Kingdom; IHMA, Schaumburg, IL

## Abstract

**Background:**

Avibactam is a β-lactamase inhibitor with potent inhibitory activity against Class A, Class C, and some Class D serine β-lactamases. The combination of ceftazidime with AVI (CZA) has been approved for several indications caused by gram-negative bacteria. This study examined the *in vitro* activity of CZA and comparators against *Pseudomonas aeruginosa* collected worldwide in 2019-2023 through the Antimicrobial Testing Leadership and Surveillance (ATLAS) program with isolates divided into those originating from adult patients (18-105 years of age [y]) and pediatric patients (0-17 y).

**Figure:**
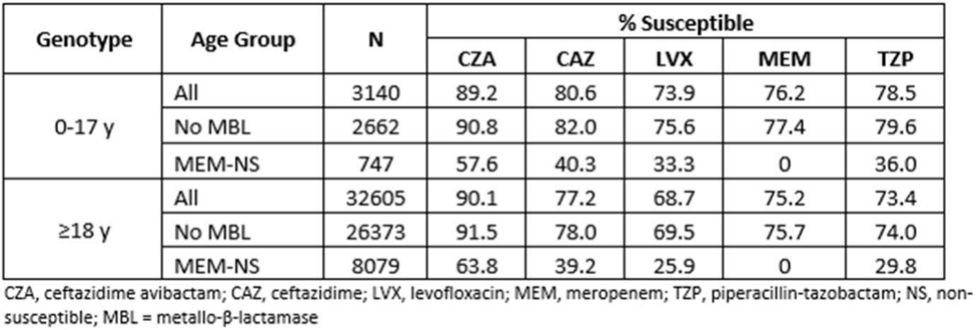

**Methods:**

35,745 non-duplicate, clinically relevant *P. aeruginosa* isolates were collected from 226 sites in 57 countries worldwide (excluding North America and China) as part ATLAS program from 2019 and 2023. Susceptibility testing was done using broth microdilution following CLSI guidelines and interpreted using CLSI 2024 breakpoints. PCR and sequencing were used to identify metallo-β-lactamase (MBL) genes among all isolates testing with meropenem MIC >1 µg/mL.

**Results:**

CZA was the most active agent examined against isolates from both pediatric and adult patients, inhibiting 89.2% and 90.1% respectively (Table).This was 13 and 15 percentage points higher, respectively, than the percentages inhibited by meropenem. Removing MBL-producers increased the percentages susceptible to CZA for both groups by 1-2 percentage points. Approximately 57-64% of the meropenem-nonsusceptible isolates from both groups were susceptible to CZA.

**Conclusion:**

CZA remains an excellent therapeutic choice for use against *P. aeruginosa*, including meropenem-nonsusceptible isolates, from all age groups.

**Disclosures:**

Katherine Perez, PhD, Pfizer: Stocks/Bonds (Public Company) Paurus Irani, MD, Pfizer, Inc.: Employee|Pfizer, Inc.: Stocks/Bonds (Private Company)

